# Levels and health risk assessment of pesticides and metals in *Lycium barbarum* L. from different sources in Ningxia, China

**DOI:** 10.1038/s41598-021-04599-5

**Published:** 2022-01-12

**Authors:** Yahong Zhang, Jiaqi Qin, Yan Wang, Tongning Zhou, Ningchuan Feng, Caihong Ma, Meilin Zhu

**Affiliations:** 1 College of Pharmacy, Ningxia Medical University, Yinchuan, 750004 China; 2 College of Public Health and Management, Ningxia Medical University, Yinchuan, 750004 China; 3 College of Basic Medical Sciences, Ningxia Medical University, Yinchuan, 750004 China; 4 College of Resources and Environmental Science, Ningxia University, Yinchuan, 750021 China

**Keywords:** Environmental sciences, Environmental chemistry

## Abstract

The berries of *Lycium barbarum* L. (Goji) are widely used as a Chinese traditional herbal medicine and functional food because of their reported beneficial pharmacological effects. However, there are reports of Goji berries being contaminated by chemical residues that could pose a hazard to humans. In this study, samples of *L. barbarum* L. berries were collected from plantations in a genuine production area and supermarkets in Ningxia, China. The major hazardous chemicals, including pesticides (dichlorvos, omethoate, cypermethrin, fenvalerate, malathion, and deltamethrin) and metals (lead (Pb), cadmium (Cd), copper (Cu), nickel (Ni), zinc (Zn), and arsenic (As)), were quantified by gas chromatography and inductively coupled plasma-optical emission spectrometry. In addition, associated daily exposures and health risks were determined using deterministic and probabilistic assessments. The levels of five pesticides from the plantation samples were considerably lower than the maximum residue limits; only dichlorvos was detected in the supermarket samples, and deltamethrin was not detected in any samples. Cu, Zn, As, Pb, Ni and Cd were detected in samples from both sources. The hazard quotient values of individual hazardous chemicals and the hazard index of combined hazardous chemicals were considerably less than 1, indicating the absence of a non-carcinogenic effect of hazardous chemical exposures through Goji berry consumption. The R value of As was much less than 10^–6^, which shows that consumption of the Goji berries had no obvious carcinogenic risks. The potentially harmful effects of the *L. barbarum* L. are more likely from berries obtained from plantations than those from supermarkets, and metal exposure is more dangerous than pesticide exposure. However, on the basis of our analysis, no population would be exposed hazardous chemicals exceeding existing standards, and the factors most affecting the health risk were exposure frequency and As content.

## Introduction

*Lycium barbarum* L. (Solanaceae), produces a fruit known as the Goji berry or wolfberry and has been used as a traditional Chinese medicine for centuries^1^. Goji berries are popular worldwide as a health food, and they are consumed in various forms, including soups, drinks, and in certain dishes^2^ because of their potential beneficial effects. Goji berries are rich in compounds with positive biological activities, including polysaccharides, carotenoids, and flavonoids^3^, and their consumption has been linked with health benefits, such as antioxidant^4^, and anticancer^5^ effects.

Goji berries are mostly cultivated in China. While, there is growing interest in Goji cultivation in other countries, for example, other East Asian countries (Japan^6^, South Korea^7^), American countries (the USA^2^, Brazil^8^), European countries (Italy^9^, Greece^10^, Poland^11^) et al. In China, the mainly cultivated areas are in northwestern provinces of China, such as Ningxia, Qinghai, Xinjiang, and Inner Mongolia, and Zhongning County in Ningxia is the geographical origin of the Goji berry. The fruit is typically orange-red (although there are black–purple varieties in Qinghai province), has a sweet taste, and matures from late June to late September. Goji berries have high sugar content and are extremely hygroscopic. In addition, they are easily damaged by worms. To eliminate pests, farmers apply pesticides during Goji growth period. Thus, Goji berries could contain pesticide residues. In addition to pesticides, Goji berries absorb metals from cultivated soil. Further, some berries are fumigated to improve their appearance, and, thus, metals also introduced by this route. Consequently, pesticides and metals are the major hazardous chemicals in Goji berries. Previously, many types of pesticides and metals have been detected in Goji berries^12,13^. According to Chinese Pharmacopoeia (2015)^9^, three pyrethroids and 12 organophosphates pesticides need to be detected in Chinese herbal medicine. However, three kinds of organophosphates pesticides are most frequently used in Goji berries by investigating the usage. Therefore, three pyrethroids pesticides (cypermethrin, fenvalerate, and deltamethrin) and three organophosphates pesticides (dichlorvos, omethoate, and malathion) were chosen in the current study. Also, according to Chinese Pharmacopoeia (2015)^14^, five metals (Pb, Cd, Cu As, and Hg) need to be detected in in Chinese herbal medicine. However, Hg was not detected in previous reports^8,15^, while Ni and Zn, which would harmful to human health in high levels, were found in our pre-experiment^16^. Therefore, six metals (Pb, Cd, Cu, As, Ni and Zn) were selected in the present study.

Numerous studies have focused on the biological activities of Goji berries, however, only a few studies have investigated pesticides and metals present Goji berries, especially with a focus on their associated health risks. Concerning pesticides, Li et al.^12^ identified 14 types of organophosphates pesticides in Goji berries using gas chromatography (GC). Huang et al.^17^ also used GC to simultaneously detect 50 kinds of organochlorine and pyrethroid pesticides in Goji berries. Chen et al.^18^ analyzed the etoxazole and pyridaben contents of Goji berries using GC method. Therefore, GC was also applied in the present study. These studies of pesticides in Goji berries did not discuss the associated health risks for consumers. While, recently, Fu et al.^19^ detected 8 pesticides and evaluated the associated dietary risk. Jing et al.^20^ analyzed 11 commonly used pesticide residues in Goji berries from Golmud area and conducted risk assessments for acute and chronic dietary exposures. Kim et al.^21^ monitored pesticides in Goji berries and assessed the short-term and highest long-term risks. However, the health risks in these studies were determined only through deterministic assessment. Considering the uncertainty of metal concentrations and the variability of exposure parameters, deterministic assessment may overestimate or underestimate risks^22^. Probabilistic assessment can solve this problem by providing probabilities and identifying priority chemicals for risk control. Therefore, both deterministic and probabilistic assessments were performed in the present work.

Several studies have reported the presence of metals in Goji berries. Sa et al.^8^ measured contents of macro- and microelements, such as Ni and Zn in Goji berries using inductively coupled plasma-optical emission spectroscopy (ICP-OES). Kulaitienė et al.^23^ also used ICP-OES method to detect metals in Goji berries. Wojcieszek et al.^13^ used inductively coupled plasma mass spectrometry (ICP-MS) to quantify different metals in Goji berries. Rangsipanuratn et al.^24^ also used ICP-MS to detect heavy metals in Chinese medicinal herbs, including Goji berries. Fu et al.^25^ measured elements in some medicine food homologous (MFH) plants, including Goji berries, by ICP-tandem mass spectrometry (MS/MS). Xu et al.^26^ used different methods to detect five toxic metals in Goji berries. The Pb and Cd contents were detected by graphite furnace atomic absorption spectrometry (GFAAS), the Cu content was determined through FAAS, and the Hg and As contents were determined by atomic fluorescence spectrometry (AFS). ICP-OES combines a wide linear range, low detection limits, good sensitivity, widespread instrument availability, and reasonable cost^8^. Therefore, in this study, ICP-OES was used to identify and quantify the metals in Goji berries. In addition, most studies have focused on optimization methods or metal levels in different parts of Goji berries and the health risk of exposure through Goji berry consumption have been neglected. Therefore, an evaluation of the health risks posed by common metals in Goji berry is essential.

In summary, considering the simultaneous exposure to the main pollutants (pesticides and metals) in Goji berries and the uncertainty of risk assessment, this study was aimed to (1) analyze the concentrations of six pesticide residues (dichlorvos, omethoate, cypermethrin, fenvalerate, malathion, and deltamethrin) and six metals (Pb, Cd, Cu, As, Ni and Zn) in Goji berries obtained from different sources in Ningxia, China; (2) determine the daily exposure to these hazardous chemicals from Goji berries; and (3) assess the health risks for consumers using deterministic and probabilistic methods.

## Results

### Levels of pesticides and metals in Goji berries

The methodological parameters for pesticides and metals were provided in Tables S1 and S2, respectively. All the parameters indicated that the methods were suitable for determining the contents of pesticides and metals.

The levels of pesticides and metals in Goji berries from different sources are summarized in Table 1. Five pesticides were detected in the Goji berry samples from plantations in a genuine production area. Meanwhile, only dichlorvos was detected in the supermarket samples. The levels of omethoate (0.02 ± 0.05 mg/kg), dichlorvos (0.02 ± 0.03 mg/kg), malathion (0.02 ± 0.03 mg/kg), cypermethrin (0.02 ± 0.03 mg/kg) and fenvalerate (0.88 ± 0.70 mg/kg) were considerably below their MRLs of 0.5, 1.0, 1.0, 0.5 and 1.5 mg/kg^27^, respectively. Deltamethrin was not detected in any sample.

**Table 1:** Levels of pesticides and metals in Goji berries (mg/kg).

Item		Level		The detection rate (%)		LOD	MRL
		Plantations (n = 37) Mean ± SD (minx-max)	Supermarkets (n = 80) Mean ± SD (minx-max)	Plantations (n = 37)	Supermarkets (n = 80)		
Pesticides	Dichlorovos	0.02 ± 0.03 (ND–0.17)	0.01 ± 0.02	21.62	10.00	0.0050	1.0^a^
	Omethoate	0.02 ± 0.05 (ND–0.16)	ND	16.22	0.00	0.0100	0.5^a^
	Malathion	0.01 ± 0.03 (ND–0.07)	ND	27.03	0.00	0.0050	1.0^a^
	Cypermethrin	0.02 ± 0.03 (ND–0.52)	ND	29.73	0.00	0.0100	0.5^a^
	Fenvalerate	0.88 ± 0.70 (ND–4.43)	ND	72.97	0.00	0.0200	1.5^a^
	Deltamethrin	ND	ND	0.00	0.00	0.0200	0.5^a^
Metals	Pb	0.35 ± 0.27 (ND–0.96)	0.08 ± 0.20 (ND–0.84)	89.19	70.00	0.0024	5.0^b^
	Cd	0.10 ± 0.07 (0.03–0.35)	0.04 ± 0.03 (ND–0.14)	100.0	72.50	0.0702	0.3^b^
	Cu	8.70 ± 2.70 (2.29–14.49)	7.55 ± 1.37 (5.96–10.03)	100.0	100.0	0.0093	20^b^
	Ni	0.88 ± 0.44 (0.21–2.52)	0.90 ± 0.57 (ND–1.51)	100.0	96.67	0.0034	0.2^c^
	Zn	19.56 ± 6.41 (10.98–35.35)	14.37 ± 4.02 (8.26–24.15)	100.0	100.0	0.0021	5.0^d^
	As	0.20 ± 0.23 (ND–0.81)	0.03 ± 0.09 (ND–0.35)	56.76	86.67	0.0075	2.0^b^

*MRL* means maximum residue limits, *ND* means not detected.

^a^Means European maximum residue limits of pesticides^27^.

^b^Means China MRLs of metals for medicinal plants and preparations^30^.

^c^Means China MRL of Ni for fruits^28^.

^d^Means China MRL of Zn for fruits^29^.

Except Ni, the contents of other five metals in Goji samples from the plantations were higher than in case of those from the supermarket. The average Ni contents were 0.88 ± 0.44 mg/kg in the plantation samples and 0.90 ± 0.57 mg/kg in the supermarket samples, and there was no significant difference in Ni content between the two groups (*p* = 0.905, > 0.05). However, there were significant differences in the Cu content (*p* = 0.031, < 0.05) and very significant differences in the contents of As, Cd, Pb, and Zn (*p* = 0.000, < 0.01). The MRL data of Ni^28^ and Zn^29^ are available for fruits but not for herbal medicines. The MRLs of Ni and Zn for fruits were applied to Goji berries because it is an MFH plant and consumed as a fruit. In such cases, the Ni and Zn levels were significantly higher than their MRLs. The average levels of Cu, Cd, As, Pb were 8.70 ± 2.70, 0.10 ± 0.07, 0.20 ± 0.23, 0.35 ± 0.27 and 7.55 ± 1.37, 0.04 ± 0.08, 0.03 ± 0.09, 0.08 ± 0.20 mg/kg in the plantation samples and supermarket samples, which were all lower than their MRL (20 mg/kg, 0.3 mg/kg, 2 mg/kg, 5 mg/kg)^30^.

### Exposure factors and daily exposures to pesticides and metals in Goji berries

The population diet survey result showed that the average daily amount of Goji berries consumed by humans is 1.37 g (DI/daily intake), the EF (exposure frequency) is 145.7 days per year, ED (exposure duration) is 5.93 year, and the BW (body weight) of consumers is 64.81 kg. Additional details are provided in Table S3. When Goji berries are used in tea or water, in this survey, the maximum dissolution rate was considered 30%^31^. The daily exposures values for pesticides and metals in Goji berries were calculated using Eqs. (2) and (3), and the results are listed in Table 2. The daily exposure values were used to calculate the health risks.

**Table 2:** Daily exposures to pesticides and metals in Goji berries (μg/[kg day]).

Pesticides	Plantations	Supermarket	Metals	Plantations	Supermarket
Dichlorovos	0.0001	0.00006	Pb	0.0030	0.0030
Omethoate	0.0002	–	Cd	0.0009	0.0007
Malathion	0.0002	–	Cu	0.0729	0.1213
Cypermethrin	0.0001	–	Ni	0.0074	0.0076
Fenvalerate	0.0074	–	Zn	0.1639	0.0637
Deltamethrin	–	–	As	0.0017	0.0003
–	–	–	As-cancer	0.00013	0.000019

– means not detected.

### Human health risk assessment

#### Deterministic health assessment

Deterministic health risks were calculated using Eqs. (2) and (3), and the results are presented in Table 3. The HQ values of individual hazardous chemicals were all less than 1, indicating the absence of a non-carcinogenic effect. For pesticides, the health risks of Goji berries from the plantations can be arranged as follows: fenvalerate > omethoate > dichlorvos > cypermethrin > malathion. The orders of health risk from metals in Goji berries from plantations and supermarkets were As > Cu > Cd > Pb > Zn > Ni and Cu > As > Zn > Ni > Cd > Pb, respectively.

**Table 3:** Non-carcinogenic risk (hazard quotient (HQ) and hazard index (HI)) of pesticides and metals and carcinogenic risk (R) of As in Goji berries.

Pesticides	Plantations (n = 37)	Supermarkets (n = 80)	Metals	Plantations	Supermarkets
Dichlorovos	3.60 × 10^–5^	1.51 × 10^–5^	Pb	0.0008	0.0002
Omethoate	6.70 × 10^–5^	–	Cd	0.0009	0.0003
Malathion	5.03 × 10^–7^	–	Cu	0.0018	0.0016
Cypermethrin	7.25 × 10^–6^	–	Ni	0.0004	0.0004
Fenvalerate	0.0004	–	Zn	0.0005	0.0004
Deltamethrin	–	–	As	0.0057	0.0008
HI_P_	0.0005	1.51 × 10^–5^	HI_M_	0.0101	0.0037
HI_total_ of plantation	0.0106		HI_total_ of supermarket	0.0037	
R_As_ of plantations	1.99 × 10^–7^		R_As_ of supermarkets	2.85 × 10^–8^	

– means no available value.

HI_P_ means HI of pesticides and HI_M_ means HI of metals.

The HI values were 0.0106 and 0.0037 for residents consuming Goji berries from plantations and supermarkets, respectively. The HI of pesticides (HI_P_) from the plantation samples was over 30 times higher than that of the supermarket samples. The HI of metals (HI_M_) from plantation samples was nearly 2.75 times than that of supermarket samples. For the Goji berries obtained from plantations, HI_P_ accounts for 4.72% of HI_total_, and HI_M_ accounts for 95.28% of HI_total_. For the Goji berries obtained from supermarkets, nearly 100% of HI_total_ was HI_M_. In summary, the Goji berries from plantations pose a greater risk than those obtained from supermarkets which can be related to the greater health risk posed by the metals than the pesticides.

The R values were 1.99 × 10^–7^ and 2.85 × 10^–8^ for customers taking Goji berries from plantations and supermarkets, respectively, which were lower than the negligible carcinogenic risks levels set by the USEPA (10^–6^). The result indicated that the carcinogenic risk posed by As of Goji berries in this study can be ignored.

#### Probabilistic health assessment

The best-fitting distributions of some EFs were simulated. For example, the metal contents in Goji berries from the plantations had beta, beta, normal, maximum extreme value, lognormal, and beta distributions for Pb, Cd, Cu, Ni, Zn, and As, respectively (Table S4). Some EFs cannot simulate the best-fitting distributions because the data size does not meet the requirements. For example, dichlorvos was detected in Goji berries from the supermarkets in only eight samples. For these exposure factors, DI, EF, ED, and BW, the distributions had beta, geometry, lognormal, and beta distributions (Table S5; Fig. S1).

The results of the probabilistic estimation of health risks are summarized in Table 4. Firstly, the probabilistic results confirm the deterministic results. Most HI values were fitted to lognormal distributions (Fig. S2). The values of the 10th, 50th, and 90th HI_P_ also suggest that the pesticides in Goji berries from plantations pose significantly higher health risks than those from the supermarkets. The 10th, 50th, and 90th HI_M_ values also indicate that the metals in Goji berries from the plantations pose a slightly higher health risk than those from the supermarkets. On the basis of the HI_total_ values, the Goji berries from plantations also pose a higher risk than those from supermarkets. Secondly, the probabilistic results indicate that a percentage of the population will be exposed to levels exceeding the standards. The results in Table 4 show that no population exceeded 1, indicating that the consumption Goji berries is safe.

**Table 4:** Statistics of the probabilistic estimation of the hazard index (HI) and R.

	Distribution	Parameters	10%	50%	90%
HI_P_ of plantations	Lognormal	Location: 0.00, mean: 5.80 × 10^–4^, SD: 1.44 × 10^–3^	2.82 × 10^–5^	2.36 × 10^–4^	1.29 × 10^–3^
HI_P_ of supermarkets	Lognormal	Location: 0.00, mean: 7.00 × 10^–5^, SD: 1.80 × 10^–4^	9.30 × 10^–7^	7.32 × 10^–6^	3.99 × 10^–5^
HI_M_ of plantations	Lognormal	Location: − 3.00 × 10^–5^, mean: 1.27 × 10^–2^, SD: 4.31 × 10^–2^	3.92 × 10^–4^	3.83 × 10^–3^	2.65 × 10^–2^
HI_M_ of supermarkets	Logic	Mean: 2.00 × 10^–3^, scaling: 2.56 × 10^–3^	4.49 × 10^–5^	1.17 × 10^–3^	8.42 × 10^–3^
R_As_ of plantations	Logic	Mean: 0.00, scaling: 0.00	− 5.80 × 10^–9^	2.41 × 10^–8^	4.96 × 10^–7^
R_As_ of supermarkets	Student T	Midpoint: 0.00, scaling: 0.00, freedom: 1	− 4.13 × 10^–8^	6.49 × 10^–10^	6.56 × 10^–8^

In the probabilistic estimation of As carcinogenic risk of Goji berries from plantations, the 50th and 90th percentile R values were 2.41 × 10^–8^ and 4.96 × 10^–7^, which were less than 10^–6^. For Goji berries from supermarkets, the 50th and 90th percentile R values were 6.49 × 10^–9^ and 6.56 × 10^–8^, which were also lower than 10^–6^. In terms of the R values, the Goji berries from plantations pose a higher risk than those from supermarkets.

### Sensitivity results

The most sensitive factors were calculated (Fig. S3). For the total non-carcinogenic risk of Goji berries from plantations, the most sensitive factors were exposure frequency (55.3%), daily intake (27.4%) of Goji berries and As content (17.4%). Concerning the total risk of Goji berries from supermarkets, the most sensitive factors were exposure frequency (48.3%), daily intake (25.3%) of Goji berries and the contents of As (24.7%), Cd (0.9%) and Pb (0.7%). In summary, exposure frequency, daily intake and As exposure were the most sensitive factors that influencing the health risk results, and control the frequency of intake is the most important way to reduce the non-carcinogenic risk of Goji berries.

For the carcinogenic risk of Goji berries from plantations, the most sensitive factors were As content (68.9%), exposure frequency (14.6%), exposure duration (8.9%), and daily intake (7.3%). For the carcinogenic risk of Goji berries from supermarkets, the most sensitive factors were As content (98.5%), exposure frequency (0.6%), exposure duration (0.5%), and daily intake (0.4%). Therefore, controlling As content is the most effective way to reduce the carcinogenic risk of Goji berries.

## Discussion

### Levels of pesticides and metals in Goji berries

Huang et al.^17^ found that cypermethrin and fenvalerate contents were 0.018–0.059 and 0.016–0.090 mg/kg, respectively, which are both remarkably lower than those in the current study. The pesticide levels were determined on the basis of many factors, such as the time and frequency of pesticide spraying and the types of pesticides. Pesticides were not detected in most supermarket samples. This result could arise for a variety of reasons. For example, some plantation samples may spray pesticides before we collect samples. Furthermore, after sale, many techniques are used to remove pesticides. Zhang et al.^32^ found that sun-drying and oven-drying can reduce pesticide residues by 10–70% and the removal rate increased as the temperature increased. The drying temperature in the present study (60 °C) was higher than that in their study (45 °C to 50 °C), so it could be speculated that the removal rate of pesticides in the present study was a litter higher. Ye et al.^33^ used ozone and alkali liquor to degrade four organophosphates pesticides and achieved a dichlorvos removal of 97.83%. Zhou et al.^34^ used a gas-phase surface discharge plasma method and verified that a large proportion of omethoate and dichlorvos (> 95%) could be removed from Goji berries. This may explain why pesticides in Goji samples from the supermarket were nearly undetectable. Goji berries are always consumed as dry berries and washed before they are eaten. In order to close the real situation, samples were washed and dried in the current study. And according to the previous reports, the pesticide residues are bound to washed off or breakdown. The exact rate of pesticides and metals washed off or breakdown should be conducted in further study.

Kai et al.^35^ collected 10 Goji samples from a planting base in Zhongning, Ningxia, and found metal contents were 0.13 mg/kg (Pb), 0.07 mg/kg (Cd), 6.00 mg/kg (Cu), 0.51 mg/kg (Ni), 16.80 mg/kg (Zn), and 0.01 mg/kg (As), respectively. Our results were 2.7 (Pb), 0.70 (Cd), 1.45 (Cu), 0.58 (Ni), and 20 (As) times higher than their results. Possible reason of the significant difference of As level was the samples were only from one planting base in their study. Wang et al.^36^ measured the heavy metals of Goji berries from peasant household in Yinchuan, Ningxia, and the contents of 4 heavy metals were 0.11 mg/kg (Pb), 0.029 mg/kg (Cd), 1.41 mg/kg (Ni), and 0.20 mg/kg (As), respectively. Their results were similar with ours. However, there are few researches on heavy metals of Goji berries from supermarkets. Some techniques, such as supercritical fluid extraction and flocculation, are used to remove metals in herbal medicines^37^. This may clarify why metals in Goji samples from the supermarket were relatively low. Furthermore, the contents of metals are varied from different areas of the world. For example, Zn, Cu, Pb, and Cd in Goji berries from Lithuania^23^, Cu and Zn levels in Goji berries from Sante (Poland)^24^, Ni, Zn and Pb levels in Goji berries from Turkey^38^, were all lower than that of our results, while As, Cd, and Pb in most Goji samples were higher than that of our results^25^. Even in the nearby areas, the results can be different. Liu et al.^39^ reported metals varied in Goji berries from different areas of Ningxia. Thus, further study of the correlation between metal contents and cultivation region is required.

### Health risk assessment

The levels of pesticides in Goji berries pose a low risk after chronic exposure in this study. Similar health risks arising from pesticides in Goji berries have been reported previously. Fu et al.^19^ found that pesticides in Goji berries do not result in health risks because the HQ is considerably less than 1. Jing et al.^20^ calculated the acute and chronic dietary exposure risks of pesticides through Goji berry consumption and found the risk to be low. Kim et al.^21^ determined the threat of pesticides in Goji berries to consumers at short-term and highest long-term exposure and no significant health risk was detected. Hence, the levels of pesticides in Goji berries do not pose obvious health risk to consumers after chronic exposure.

Concerning the health risk assessment of metals, no other investigations on Goji berries are available. We previously studied other MFH plants and found that the presence of metals in these types of plants does not pose non-carcinogenic health risks (Table 5). For example, the HI values of metals in *Panax notoginseng*^40^, *Glycyrrhizae radix*^41^, *Astragalus membranaceus*^42^, and *Prunella vulgaris*^43^ were 0.013, 0.042, 0.049, and 0.029, respectively, which are considerably less than 1.

**Table 5:** The non-carcinogenic health risk and carcinogenic risk of metals in medicine food homologous plants.

Medicine food homologous plants	Non-carcinogenic risk (HI)	Carcinogenic risk (R)	References
*Lycium barbarum* L.	0.0106 (plantations)	1.99 × 10^–7^ (plantations)	The present study
	0.0037 (supermarkets)	2.85 × 10^–8^ (supermarkets)	
*Panax notoginseng*	0.013	2.1 × 10^–6^	Zhu et al.^40^
*Glycyrrhizae radix*	0.042	4.11 × 10^–6^	Zhu et al.^41^
*Astragalus membranaceus*	0.049	4.3 × 10^–6^	Tian et al.^42^
*Prunella vulgaris*	0.029	–	Cao and Zhu^43^
Yam	–	5.9 × 10^–5^	Zhu et al.^44^
Haw	–	2.4 × 10^–5^	Zhu et al.^44^
Jujube	–	8.3 × 10^–7^	Zhu et al.^44^

– means no available data.

The result indicated that the carcinogenic risk posed by As of Goji berries in this study can be ignored. However, the carcinogenic risk posed by As is always higher than the negligible carcinogenic risk level (10^–6^) but lower than the maximum acceptable carcinogenic risk level (10^–4^) (Table 5). For example, the *R* values of As in *Panax notoginseng*^40^, *Glycyrrhizae radix*^41^, *Astragalus membranaceus*^43^, yam, and haw^44^ are 2.1 × 10^–6^, 4.11 × 10^–6^, 4.3 × 10^–6^, 5.9 × 10^–5^, and 2.4 × 10^–5^, respectively. The carcinogenic risk value of As in jujube^44^ is 8.3 × 10^–7^, which is far less than the negligible level (Table 5).

Goji berries are mostly taken as an herbal medicine, the ADI of hazardous chemicals from medicines (ADI-_medicine_) should not exceed 1% of ADI, as suggested by the WHO^45^, and the factors of DI, EF, ED are also totally different. Assuming this condition, the exposure values, HQ of individual hazardous chemicals, and HI of combined hazardous chemicals are different from the present values which should be verified in further study. In addition to Goji berries, many other foods, such as rice, vegetables, and fruits, expose residents to pesticides and metals^46–48^. Considering daily dietary intake, total health risk should not be disregarded. Moreover, ingestion methods, such as direct consumption, dissolution in water, and use as medicine, will seriously affect the results. Thus, these factors should be the focus of future studies.

In summary, it’s more accurate to evaluate health risk caused by main chemical contaminates (pesticides and metals) in Goji berry simultaneously and assess health risk by probabilistic methods considering the uncertainty. Furthermore, the related ways of controlling risk are provided. However, there are still some limitations, the removal rate of pesticides and metals by washing and drying, the correlations between metals and regions, the health risk considering more factors should be investigated in further study.

## Methods

### Sample collection and laboratory pretreatment

**Figure 1:**
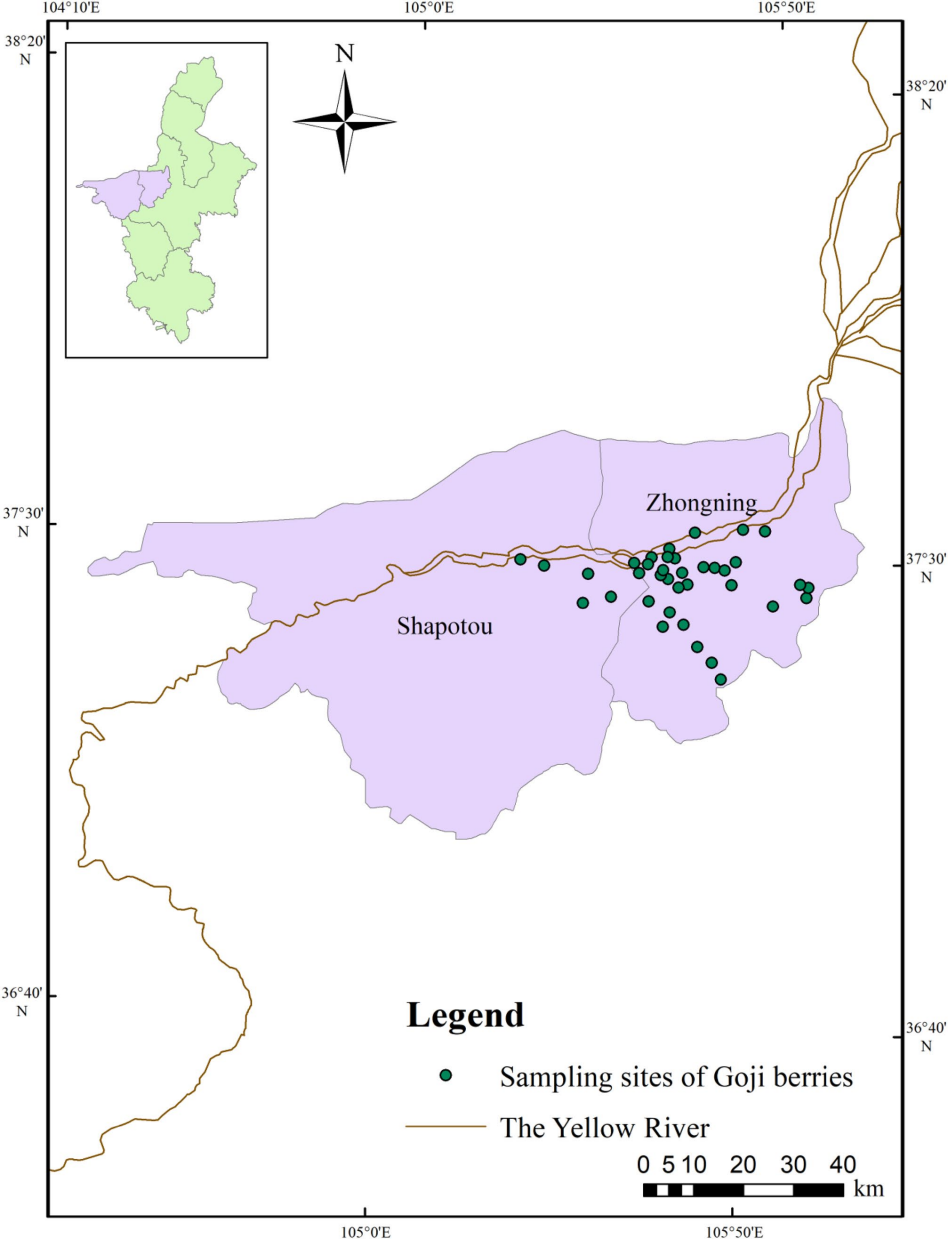
Sampling sites of Goji berries from plantations in genuine producing area (represents sampling sites) (data source: http://bbs.3s001.com/forum.php?mod=viewthread&tid=133741&page=1#pid2930761).

Goji samples (approximately 500 g when fresh) were collected from 37 plantations in Zhongning County (genuine production area) in Ningxia Province, China in June 2020, as shown in Fig. 1. All the Goji berries were cultivated by local famers. In each plantation, five subsamples were collected. The Goji samples were packed into bags, marked, immediately transported to the laboratory, and stored at 4 °C. 80 commercial Goji samples were purchased from local 15 supermarkets in Yinchuan, Ningxia, China. 5–6 samples at different prices were obtained in each supermarket. All Goji samples were identified by Professor Liming Zhang, School of Pharmacy, Ningxia Medical University.

In order to better simulate the actual situation of Goji berries intake, the Goji samples were first washed with tap water and, then, with deionized water. Subsequently, they were dried in an oven (DHG-9030A, China) at 60 °C for approximately 5 days (the moisture was less than 13%). Finally, the dried samples were ground using high-speed universal pulverizer (FW100, China), sifted through a 60-mesh sieve, and stored in sealed sample bags until analysis.

### Pesticide determination

Pesticide determination was carried out with reference to the 2015 edition of *China Pharmacopoeia*^9^, “Determination of pesticide residues.” Three pyrethroids (cypermethrin, fenvalerate, and deltamethrin) and three organophosphates (dichlorvos, omethoate, and malathion) pesticides were detected by gas chromatograph (GC) (Agilent 7890B, USA) method. The chromatographic conditions for GC analysis for pesticides detection are summarized in Table S5. Three replicates were performed to ensure precision. Standard curves were drawn using data obtained from a series of standard solutions. A blank control, as well as recovery test, was used to ensure accuracy. The limits of detection (LOD) and limits of quantification (LOQ) of each hazardous chemical were calculated with signal-to-noise (S:N) ratios of 3:1 and 10:1, respectively.

#### Metal determination

The determination of metals in Goji berries has been reported previously^16^. First, 0.2 g dried Goji berry samples were digested using a mixture of HNO_3_ and HClO_4_ (4:1, *V:V*) in low temperature combined digester (ED54-iTouch, China), and the six metals were quantified via ICP-OES (Varian 710-ES, USA). Three replicates were performed to ensure precision. Standard curves were drawn using a series of standard solutions. A blank control, a recovery test, and a standard reference were employed to ensure accuracy. The LOD and LOQ were also calculated. The conditions for the analysis of metals are provided in Table S7.

### Population diet survey

#### Determination of sample size

According to the principle of random sampling, representative objects of edible Goji berries from a producing area were selected; the sample size was calculated according to Eq. (1):

1 $$\mathrm{n}=\frac{{u}^{2}\times P(1-P)}{{E}^{2}},$$

where n is the sample number, *u* is the critical value corresponding to a certain degree of confidence, *P* is the dispersion degree of the sample, and *E* is the sampling error range. The confidence level was set to 95%, so *u* was 1.96, and *E* was ± 5%. The sample number was the largest when *P* was 0.5, and the calculated sample size was 384.

#### Questionnaire on Goji consumption

To determine the Goji berry consumption habits of consumers, questionnaire was conducted in the cities of Zhongning and Yinchuan in Ningxia, China in June 2020. A total of 712 inhabitants, were asked to join our survey and 558 respondents participated in our face-to-face interview or online questionnaire eventually with the response rate of 78.37%. According to expert’s recommendation, the participants who consumed Goji berries at least once a week and last for three months or more were taken into account of chronic exposure. They were asked to recall the quantity, frequency, duration, and way of their consumption of Goji berry, as well as to provide their age and body weight. 401 valid questionnaires were collected. The Goji berry consumption information was used for health risk assessment.

### Daily exposure

Goji berries are typically consumed by adults as a health product. The daily exposure to pesticides and metals through Goji berry consumption was calculated using Eqs. (2) and (3) (US Environmental Protection Agency)^49^:

2 $$EXPO=\frac{C\times DI\times EF\times ED}{BW\times AT},$$

3 $${EXPO}_{As}=\frac{c\times DI\times EF\times ED}{BW\times LT},$$

Equation (2) was used to calculate non-carcinogenic exposure and Eq. (3) was to calculate As carcinogenic exposure. Here, EXPO (μg/[kg day]) represents the exposure to hazardous chemicals, C (mg/kg) is the concentration of hazardous chemicals in Goji berries, DI (g/day) is the daily intake of Goji berries, EF (day/year) is the exposure frequency, ED (year) represents the exposure duration, BW (kg) is the body weight of residents, AT (year) is the average exposure time (ED × 365 day/year), LT (year) is the average lifespan of the consumers and is 76.34^50^.

### Health risk assessment

#### Deterministic assessment

All six pesticides exhibit non-carcinogenic effects through chronic oral exposure. Cd, Pb, Cu, Ni, and Zn pose non-carcinogenic health risks, and As poses non-carcinogenic and carcinogenic health risks. Non-carcinogenic individual hazardous chemical levels can be assessed by estimating the hazard quotient (HQ) value, which can be calculated using Eq. (4)^49^:

4 $$HQ=EXPO/ADI,$$

here, EXPO (μg/[kg day]) represents the exposure to hazardous chemicals, and ADI (μg/[kg day]) is the allowable daily intake. The values of each hazardous chemical are summarized in Table 6. The ADI values for six pesticides were set by the World Health Organization (WHO)^51^, and the ADI values for six metals were suggested by the USEPA^52^. The ADIs of the pesticides were 4, 3, 300, 20, 20, and 10 μg/[kg day] for dichlorvos, omethoate, malathion, cypermethrin, fenvalerate, and deltamethrin, respectively. The ADIs of Pb, Cd, Cu, Ni, Zn, and As were 3.6, 1, 40, 20, 300, and 0.3 μg/[kg day], respectively. When the interactions between hazardous chemicals are not considered, the hazard index (HI; Eq. 5) can be calculated to evaluate the total non-carcinogenic health risk upon exposure to more than one hazardous chemical^53^,

**Table 6:** Allowable daily intake (ADI) of pesticides and metals (μg/[kg day]).

Pesticides	ADI (μg/[kg day])	Metals	ADI (μg/[kg day])
Dichlorovos	4	Pb	3.6
Omethoate	3	Cd	1
Malathion	300	Cu	40
Cypermethrin	20	Ni	20
Fenvalerate	20	Zn	300
Deltamethrin	10	As	0.3

ADI of pesticides^51^.

ADI of metals^52^.

5 $$HI = {\sum }_{1}^{n}{HQ}_{n}.$$

The carcinogenic risk (*R*) posed by As was evaluated using Eq. (6)^54^,

6 $$R=SF\times EXPO,$$

here, SF is the slope factor with a suggested value of 1.5 mg/[kg day]^55^.

#### Probabilistic assessment

Average values are used in deterministic assessment; but they cannot reflect the uncertainty and variability in health risk evaluation. Consequently, probabilistic assessment was used to assess the probabilistic distribution of daily exposure to hazardous chemicals and harmful risks. Monte Carlo simulation is widely used in probabilistic estimation^56^ and can be described as follows. First, the best-fitting distributions of exposure parameters are obtained by fitting several parametric distributions (e.g., lognormal, gamma, and beta) and selecting the distribution with the best statistical results, such as the Anderson–Darling test and chi-square (*χ*^*2*^) test. Then, exposure parameter values are randomly selected from the best distribution and simulated with thousands of iterations to obtain stable distributions of exposures and health risks. The results of probabilistic estimations are frequently presented in distribution, distribution parameters, and percentile values (e.g., 10th, 50th, and 90th). The entire process was carried out using Crystal Ball.

### Statistical methods

Mean and standard deviation (SD) were calculated in Microsoft Excel 2010 (Microsoft Ins., USA). The Mann–Whitney U test was conducted in SPSS 21.0 (IBM Ins., USA). The determination of the best-fitting distribution and the Monte Carlo simulation were performed using Crystal Ball software (Oracle© Ins., USA). ArcGIS Desktop 10 (ESRI Ins., USA, Authorization number: EFL564098460) was used to map the sampling sites.

### Ethics approval and consent to participate

The collection of Goji samples from plantations was permitted by local famers orally. The plant collection and the study complied with local (Ningxia) and national (China) regulations. The study was approved by the institutional research ethics committee of Ningxia Medical University, and written informed consent was obtained from each participant. All the methods in this manuscript were carried out in accordance with relevant guidelines and regulations.

## Supplementary Information

Supplementary Information.

## Data Availability

The datasets used and/or analyzed during the current study available from the corresponding author on reasonable request.
