# Supplementary material for: Levels and health risk assessment of pesticides and metals in Lycium barbarum L. from different sources in Ningxia, China
Source: Sci Rep. 2022 Jan 12;12:561. doi: 10.1038/s41598-021-04599-5 (PMC8755795; doi:10.1038/s41598-021-04599-5)
Supplement: Supplementary file 1 — Supplementary Information. [file 41598_2021_4599_MOESM1_ESM.docx]

**Levels and health risk assessment of pesticides and metals in** ***Lycium barbarum* L.** **from different sources in Ningxia, China**

Yahong Zhang^1^, Jiaqi Qin^2^, Yan Wang^2^, Tongning Zhou^2^, Ningchuan Feng^1,3^, Caihong Ma^4^, Meilin Zhu^1,2,3*^

^1^College of Pharmacy, Ningxia Medical University, Yinchuan 750004, China

^2^College of Public Health and Management, Ningxia Medical University, Yinchuan 750004, China

^3^College of Basic Medical Sciences, Ningxia Medical University, Yinchuan 750004, China

^4^College of Resources and Environmental Science, Ningxia University, Yinchuan 750021, China

* Correspondence: Prof. Meilin Zhu, E-mail: jay70281@163.com, Tel: 0951-6980120

**List of Supplementary Data**

Figure S1 Distribution of various parameters

Figure S2 Forecast result of the metals

Figure S3 Sensitivity result of the metals

Table S1 The methodological verification of pesticide detection

Table S2 The methodological verification of metal detection

Table S3 The result of questionnaire on Goji consumption

Table S4 The probabilistic distribution of metal contents

Table S5 The probabilistic distribution of exposure factors

Table S6 The operating parameters of GC analysis to detect pyrethroids and organophosphates pesticides

Table S7 ICP-OES working parameters


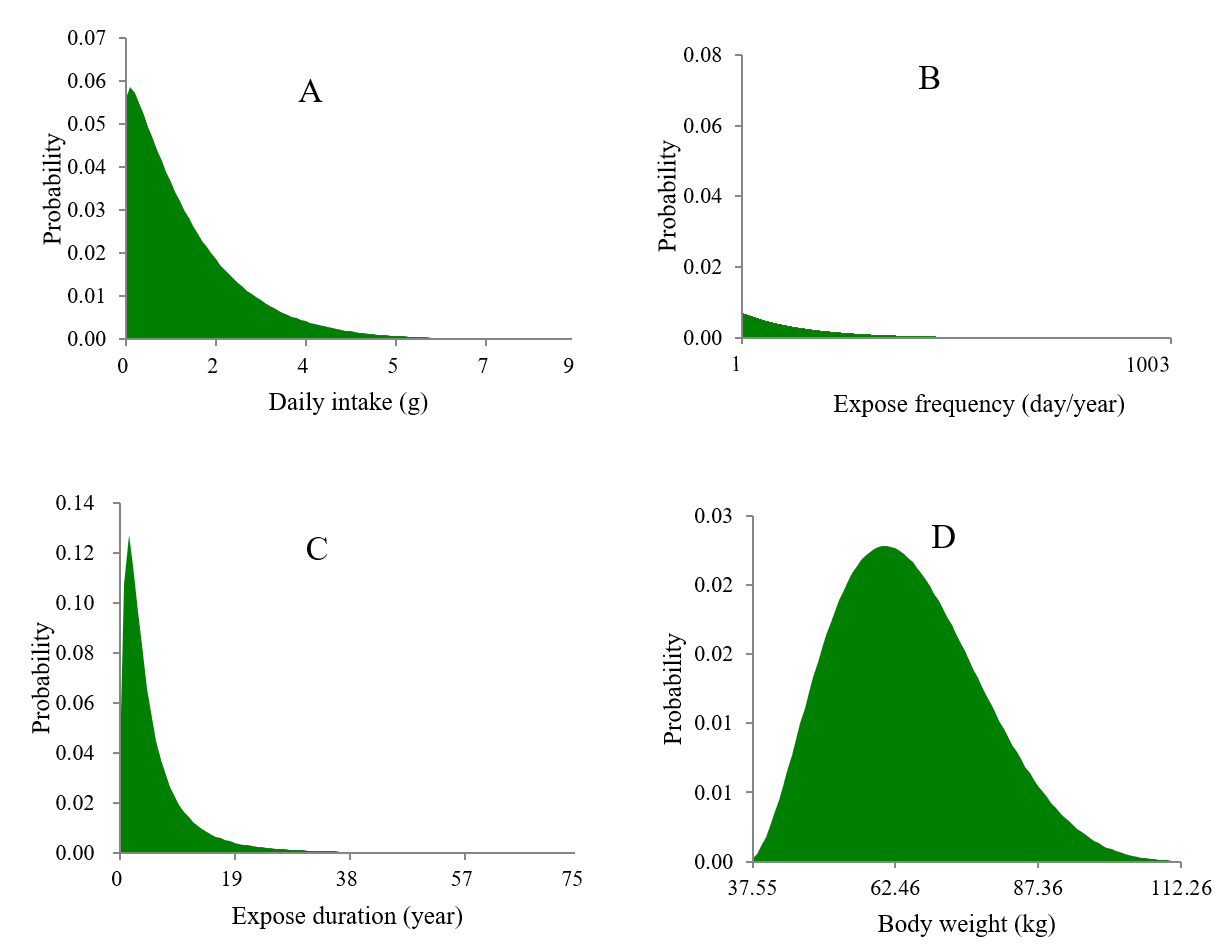


A. Distribution of daily intake (g)

B. Distribution of expose frequency (day/year)

C. Distribution of expose duration (year)

D. Distribution of body weight (kg)

**Figure S1** Distribution of various parameters


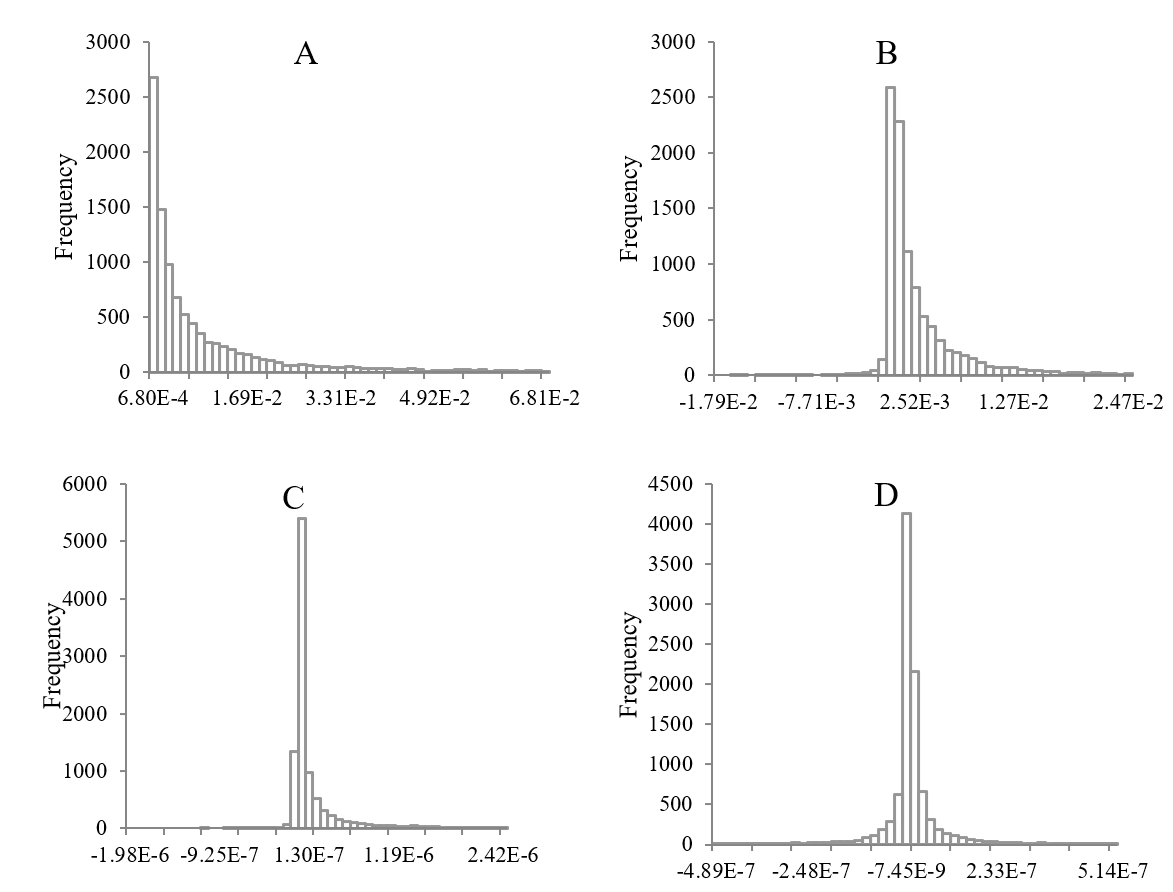


A. Forecast of HI in Goji from plantation

B. Forecast of HI in Goji from supermarket

C. Forecast of R in Goji from plantation

D. Forecast of R in Goji from supermarket

**Figure S2** Forecast result of the metals


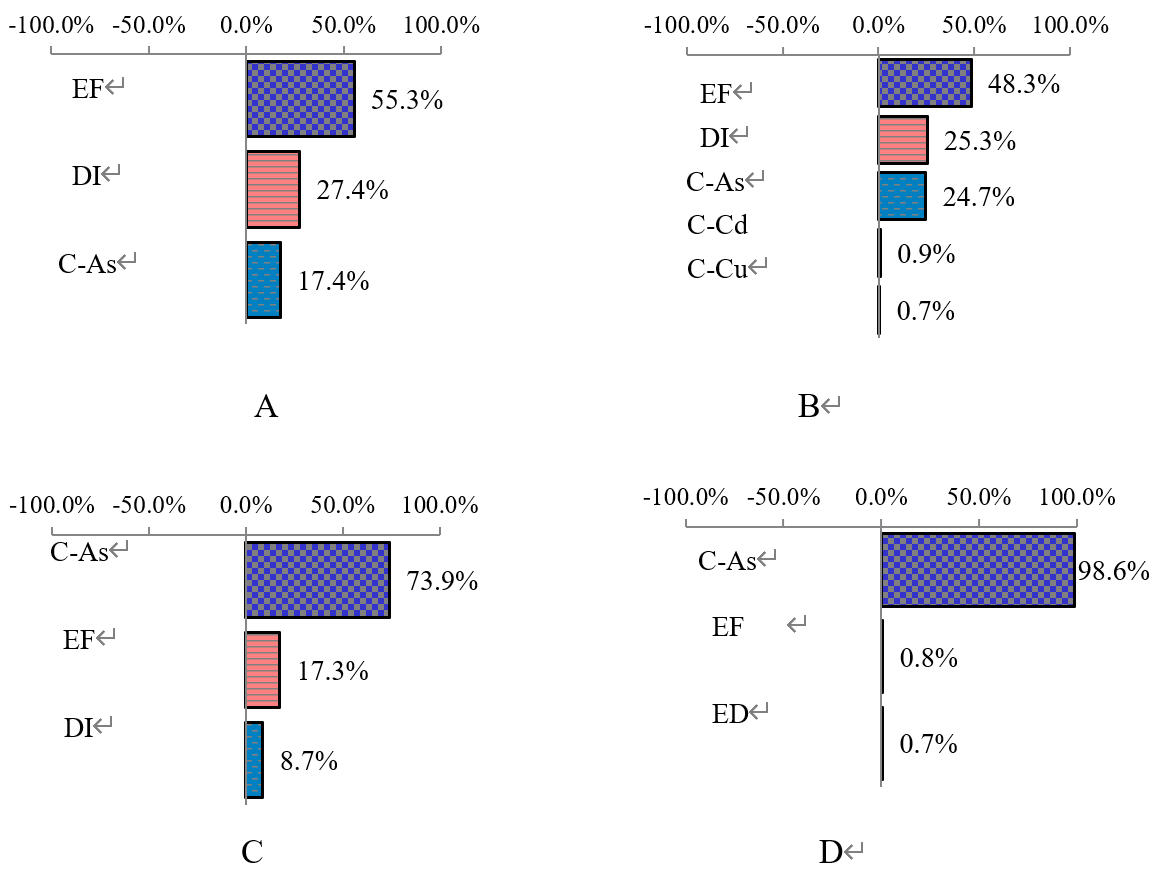


A. Sensitivity result of HI-metals Goji from plantation

B. Sensitivity result of HI-metals Goji from supermarket

C. Sensitivity result of R-As Goji from plantation

D. Sensitivity result of R-As Goji from supermarket

**Figure S3** Sensitivity result of the metals

**Table S1** The methodological verification of pesticide detection

| Pesticides | RT  (min) | Linear regression equations | correlation coefficients | Recovery (%) | RSD (%) | LOD  (µg/g) | LOQ  (µg/g) |
| --- | --- | --- | --- | --- | --- | --- | --- |
| dichlorovos | 4.47 | y=4.1824x-0.5483 | 0.9979 | 77.25 | 4.05 | 0.0050 | 0.0150 |
| omethoate | 7.67 | y=0.1892x-0.0375 | 0.9960 | 86.14 | 5.73 | 0.0100 | 0.0300 |
| malathion | 11.29 | y=0.3980x-0.0325 | 0.9971 | 92.37 | 3.06 | 0.0050 | 0.0150 |
| cypermethrin | 8.00 | y=11.068x-0.4788 | 0.9940 | 90.02 | 4.52 | 0.0100 | 0.0300 |
| fenvalerate | 9.81 | y=4.5172x-0.6651 | 0.9967 | 79.91 | 3.27 | 0.0200 | 0.0600 |
| deltamethrin | 11.45 | y=8.5566x-2.2726 | 0.9990 | 91.14 | 7.03 | 0.0200 | 0.0600 |

RT: retention time

LOD: limit of detection

LOQ: limit of quantitation

**Table S2** The methodological verification of metal detection

| Element | Linear regression equations | correlation coefficients | Recovery (%) | RSD (%) | LOD  (µg/g) | LOQ  (µg/g) |
| --- | --- | --- | --- | --- | --- | --- |
| Pb | y=2055.4x+101.34 | 0.9999 | 80 | 6.37 | 0.0024 | 0.0072 |
| Cd | y=33932x+531.04 | 1 | 106 | 9.16 | 0.0702 | 0.2106 |
| Cu | y=16204x+657.2 | 1 | 91 | 1.12 | 0.0093 | 0.0279 |
| Ni | y=6400.1x+273.56 | 1 | 102 | 3.44 | 0.0034 | 0.0102 |
| Zn | y=21018x+1643.1 | 0.9999 | 99 | 5.23 | 0.0021 | 0.0063 |
| As | y=525.5x+3.7121 | 1 | 105 | 5.29 | 0.0075 | 0.0225 |

LOD: limit of detection

LOQ: limit of quantitation

**Table S3** The result of questionnaire on Goji consumption

| Gender | | Age (year) | | | | | Average weight (kg) | Average daily intake (g) | Average exposure frequency (day/year) | Average exposure duration (year) |
| --- | --- | --- | --- | --- | --- | --- | --- | --- | --- | --- |
| Male (number) | Female (number) | <20 (number) | | 20-40 (number) | 40-60 (number) | >60 (number) |  |  |  |  |
| 206 | 95 | 2 | 152 | | 212 | 35 | 64.81 | 1.37 | 145.7 | 5.93 |

**Table S4** The probabilistic distribution of metal contents

| Element | Distribution types |
| --- | --- |
| Pb | Beta |
| Cd | Beta |
| Cu | Normal |
| Ni | Maximum extreme value |
| Zn | Lognormal |
| As | Beta |

**Table S5** The probabilistic distribution of exposure factors

| Exposure factors | Distribution types |
| --- | --- |
| Daily intake | Beta |
| Expose frequency | Geometry |
| Expose duration | Lognormal |
| Body weight | Beta |

**Table S6** The operating parameters of GC analysis to detect pyrethroids and organophosphates pesticides

| Item | Parameters | |
| --- | --- | --- |
|  | Pyrethroids pesticides | Organophosphates pesticides |
| Detector | Electron capture detector (ECD) | Flame photometric detector (FPD) |
| Chromatographic column | HP-5 | HP-5 |
| Carrier gas | N_2_ | N_2_（9 ml/min）H_2_（40 ml/min）  Air (150ml/min) |
| Injection port temperature | 260℃ | 220℃ |
| Detector temperature | 300℃ | 300℃ |
| Injection volume | 1 µl | 1 µl |
| Injection method | Split-flow injection (1:5) | No-split-flow injection |
| Temperature | The initial temperature was 260℃(9min), maintained for 5 min, and then increased to 270℃ with the rate of 2℃/min and maintained for 5 min | The initial temperature was 120℃(9min), and then increased to 240℃ with the rate of 10℃/min and maintained for 2 min |

**Table S7** ICP-OES working parameters

| Parameters | Numerical value |
| --- | --- |
| Power (KW) | 1.00 |
| Plasma gas flow (L·min^-1^) | 15.0 |
| Auxiliary gas flow (L·min^-1^) | 1.50 |
| Nebulizer pressure (kPa) | 200 |
| One reading time (s) | 5 |
| Pump speed (rpm) | 15 |
| Cleaning time (s) | 10 |
| Condition | All spectral lines |
